# Translation, cultural adaptation, and psychometric evaluation of the arabic version of the type 1 diabetes stigma assessment scale (DSAS-1-Ar) among adults in Jazan, Saudi Arabia

**DOI:** 10.1007/s40200-026-02022-2

**Published:** 2026-07-24

**Authors:** Husameldin Elsawi Khalafalla, Osama Albasheer, Fatima A. Elfaki, Abdulrahman Hummadi, Yahia Solan, Rania Hassan, Norah Rajeh, Taif Solan, Huda Mohamed Haroun Adam, Stef P. J Kremers

**Affiliations:** 1 Research Institute of Nutrition and Translational Research in Metabolism (NUTRIM), Department of Health Education and Promotion, Faculty of Health, Medicine & Life Sciences, Maastricht University, Maastricht, The Netherlands; 2 Department of Family and Community Medicine, Jazan University, Jazan, Saudi Arabia; 3 Department of Clinical Nutrition, College of Nursing and Health Sciences, Jazan University, Jazan, Saudi Arabia; 4 Adult Endocrinology and Diabetes Department, Jazan Endocrinology & Diabetes Center, Ministry of Health, Jazan, 82723 Saudi Arabia; 5 Faculty of Medicine, MBBS, Jazan University, Jazan, Saudi Arabia; 6 Faculty of Medicine, University of Gezira, Medani, Sudan

**Keywords:** Type 1 diabetes, Stigma, DSAS-1, Arabic validation, Psychometrics, Saudi Arabia

## Abstract

**Background:**

Diabetes-related stigma adversely affects psychological wellbeing, self-care behaviors, and glycemic control in individuals with Type 1 diabetes (T1D). Despite the high prevalence of T1D in the Middle East and North Africa region, validated Arabic instruments to assess stigma remain limited. This study aimed to translate, culturally adapt, and evaluate the psychometric properties of the Arabic version of the Type 1 Diabetes Stigma Assessment Scale (DSAS-1-Ar) from the original English instrument.

**Methods:**

A cross-sectional study was conducted among 299 adults with T1D attending the Endocrinology and Diabetes Centre in Jazan, Saudi Arabia, recruited using consecutive sampling (response rate 99.3%). Translation followed established cross-cultural adaptation guidelines. Construct validity was examined using confirmatory factor analysis, with three competing structural models tested. In addition, Known-groups comparisons were executed between DSAS-1-Ar scores and self-reported clinical variables.

**Results:**

All 19 items loaded significantly onto their hypothesized latent factors (Treated Differently (TD), Blame and Judgment (BJ), and Identity Concerns (IC); standardised loadings 0.642–0.947). Internal consistency was good (total α = 0.985, ω = 0.986). None of the three compared structural models met conventional fit thresholds (CFI range 0.812–0.888; RMSEA range 0.167–0.202). SRMR for the three-factor model was acceptable (0.065), while CFI (0.816) and RMSEA (0.202) indicated suboptimal global fit. Known-groups validation analyses yielded plausibly different scores across distinct populations; e.g. higher stigma scores among females, older participants and those with diabetes-related complications.

**Conclusions:**

The DSAS-1-Ar represents an important initial step toward assessing T1D-related stigma in Arabic-speaking populations. Items loaded strongly onto theoretically assigned subscales and internal consistency was good. However, global CFA fit indices were suboptimal, indicating that the factor structure requires further evaluation in this cultural context. Future studies can include assessing convergent validity, test-retest stability, and model fit in more diverse Arabic-speaking samples.

**Trial registration:**

Clinical trial number: not applicable.

**Supplementary Information:**

The online version contains supplementary material available at 10.1007/s40200-026-02022-2.

## Introduction

Type 1 diabetes (T1D) is a chronic disease in which autoimmune processes lead to progressive destruction of the insulin-producing beta-cells of the pancreas, resulting in insulin deficiency. Although T1D can develop at any age, the onset of overt disease is most frequent in children and young adults [1].

The Middle East and North Africa (MENA) region, which includes many Arabic-speaking countries such as Saudi Arabia, contains some of the highest diabetes prevalence rates globally, with a projected 92% increase by 2050 [1], making diabetes a regional health priority. In the Kingdom of Saudi Arabia (KSA), diabetes is a major public health problem. The Kingdom ranks among the highest 10 countries regarding the prevalence of diabetes [1]. The number of children living with diabetes in KSA in the year 2024 was estimated at 46,000, placing KSA among the top five countries with the highest prevalence globally [1].

Type 1 diabetes places a substantial burden not only on individuals living with the condition, but also on their caregivers and the broader healthcare system. This chronic condition imposes lifelong daily demands, including lifestyle adaptations and the administration of insulin either by injections or pumps along with glucose monitoring through multiple daily tests or continuous monitoring. These ongoing responsibilities often lead to emotional strain and a diminished quality of life [2]. This burden emphasizes the importance of creating environments that enable effective self-care and long-term disease control. To achieve this, it is essential to remove not only clinical and logistical obstacles but also social and psychological barriers including those imposed by diabetes-related stigma.

Diabetes-related stigma (DRS) refers to “negative social judgments, stereotypes, and prejudices about diabetes, or about a person or group due to their diabetes, occurring typically in the context of a power imbalance” [2] (p. 62). Stigma types include felt stigma (perceived by the individual), enacted stigma (experienced through others’ actions), or internalized stigma (when people with the condition become preoccupied with others’ opinions and accept negative judgments) [2–4]. Intersectional stigma occurs when DRS is combined with other stigmatized conditions or characteristics such as obesity or race [2].

While both T1D and Type 2 Diabetes (T2D) patients may encounter stigma, it can be assumed that individuals with T1D are more prone to stigma due to the greater visibility of their condition. The requirement for multiple daily insulin injections or pumps, frequent blood glucose monitoring, and the higher risk of hypoglycemic episodes are more commonly seen in T1D. These observable aspects of self-management may draw public attention and scrutiny [5]. Despite challenges in measurement of DRS stemming from the absence of a unified tool and standardized cutoff points stigma related to T1D is likely widespread. In a systematic review, Eitel et al. [6] reported prevalence rates ranging from 52% to 78% in adults with T1D and 59% to 98.7% in youth and adolescents with T1D.

The negative effects of DRS have become a growing concern in global diabetes care and research [7, 8]. For people living with diabetes, stigma is accompanied by many negative experiences. They encounter blame, judgment, exclusion, feelings of guilt and shame, which can lead to harmful emotional outcomes such as self-blame, anxiety, and isolation [9, 10]. DRS is strongly associated with challenges to mental health, including distress, anxiety, diabetes-related distress, and depression [11, 12]. In response to these experiences, many patients choose to conceal their diabetic condition [5]. This concealment often leads to suboptimal self-care behaviors, such as avoiding glucose monitoring, delaying insulin injections, or not adhering to dietary plans in public [5]. These disruptions in self-management can, in turn, lead to poorer clinical outcomes. Several studies have shown a consistent association between DRS and elevated glycated hemoglobin (HbA1c) levels, underscoring the significant impact of stigma on both psychological wellbeing and physical health [2, 13].

Motivated by the significant impact of DRS on diabetes management and the wellbeing of people with diabetes, and noting the absence of suitable measurement tools, Browne et al. [14] created and validated the Diabetes Stigma Assessment Scale-1 (DSAS-1). The rigorous development of this self-reported questionnaire began with an exploratory qualitative study to generate an initial pool of 64 items, which were then reduced to 19 through a psychometric validation process. Known-groups analyses and clinical correlations have indicated support for the construct validity of the translated scale [15].

The growing importance of diabetes-related stigma, coupled with the scarcity of research, especially on T1D [5] and the limited evidence from the MENA region, underscores the need to develop appropriate tools to explore this phenomenon. While global research on DRS is gaining momentum, most existing studies have focused on T2D [5] and on non-Arabic speaking countries. The aim of this study is to provide researchers in Arabic-speaking populations access to a tool tested for validity and reliability in T1D patients from Saudi Arabia.

## Materials and methods

### Translation

The translation and cultural adaptation process followed the methodology proposed by Beaton et al. (2000) with additional steps to enhance linguistic and clinical validity [16]. The process conformed with the guidelines of the Mapi Research Trust, the copyright holders.

### Forward translation

Two independent bilingual English language teachers with Arabic as native language from the University of Jazan independently performed forward translations into Arabic. Discrepancies were reconciled in a meeting with the translators facilitated by the principal investigator, producing a single harmonized version.

### Backward translation

The harmonized version was independently back-translated into English by an assistant professor and an associate professor with teaching experience in English-language instruction and Arabic as native language, blinded to the original questionnaire. Discrepancies between the original version and the back-translation were reviewed.

### Proofreading

The pre-final Arabic version was reviewed by experts at the Khartoum University Language Centre (Sudan) for grammatical accuracy and fluency.

### Expert committee review

After being reviewed for clinical and conceptual soundness by 3 of the authors with extensive experience in managing diabetes patients, two external family physicians reviewed the questionnaire for clinical appropriateness, idiomatic accuracy, and cultural equivalence and acceptability, reaching consensus on the final version.

### Cognitive interviewing

Cognitive interviews were conducted with 13 participants (9 males, 4 females; age range 21–79 years) consecutively selected from the target population on the day of data collection. The interviews explored the clarity, relevance, and cultural appropriateness of each item and were documented in written notes by the data collectors. Feedback from this phase led to minor revisions, resulting in the final Arabic version of the questionnaire.

### study Design, Study Area and Population

Inclusion criteria: (1) confirmed diagnosis of Type 1 diabetes; (2) age ≥ 18 years; (3) attending the EDC in Jazan; (4) ability to read and respond to Arabic text.

Participants were adults with Type 1 diabetes attending the Endocrinology and Diabetes Centre (EDC) in Jazan city, located in the southwest of the Kingdom of Saudi Arabia, capital of Jazan Province.

Participants were recruited using consecutive sampling. Of 301 eligible clinic attendees invited to participate during the study period, 299 consented to enrol (response rate 99.3%); 2 individuals declined participation. No participants were excluded after enrolment. All participants who attended the clinic in the period from September–November 2024 were invited to participate.

### Data collection

In total, 299 consenting participants completed a self-administered questionnaire. Data collectors were trained to administer and explain any item that might require clarification. Assistance with reading items was available to participants who required it. For participants who were unable to read, items were read aloud by trained data collectors, and responses were recorded as stated by the participant.

No items were missing in the final dataset; all 299 participants completed all 19 DSAS-1-Ar items.

### Scale description

The original DSAS-1 questionnaire was developed and validated by Browne et al. [14]. Three factors constitute the 19-item tool:

1. Treated Differently (TD, 6 items): captures experiences of being discriminated against or excluded due to diabetes.
2. Blame and Judgment (BJ, 6 items): reflects perceptions of being blamed for having diabetes or judged negatively.
3. Identity Concerns (IC, 7 items): reflects perceived threats to self-identity or concerns about being misidentified due to diabetes.

Each item is scored on a 5-point Likert scale (1 = Strongly Disagree to 5 = Strongly Agree). Higher scores indicate greater perceived stigma. Subscale scores are the sum of constituent items (BJ range: 6–30; IC range: 7–35; TD range: 6–30; Total range: 19–95).

### Data analysis

With *N* = 299 participants and 19 free parameters in the three-factor Confirmatory factor analysis (CFA) model, the participant-to-parameter ratio is approximately 15.7:1, exceeding the recommended minimum of 10:1 for CFA [17].

Descriptive statistics were calculated using SPSS v26. Reliability analysis included Cronbach’s alpha, McDonald’s omega, corrected item-total correlations, and alpha-if-item-deleted statistics for each subscale and the total scale.

Confirmatory factor analysis (CFA) and known-groups validity analyses were conducted using R version 4.5.0 [18] and the lavaan package [19]. Known-groups comparisons used Mann–Whitney U tests with rank-biserial correlation as an effect size measure. Spearman’s rho examined associations between DSAS-1-Ar scores and clinical variables (HbA1c, Fasting Blood Glucose).

### Confirmatory factor analysis (CFA)

CFA was conducted to assess construct validity. A three-factor model was specified a priori, reflecting the original structure of the scale. The models were built using maximum likelihood with robust standard errors (MLR), which provides Satorra–Bentler scaled test statistics that correct for non-normality and has been shown to perform adequately for 5-point Likert items with samples exceeding *n* = 200 [20]. Three competing models were tested: (M1) a single-factor model; (M2) the original three-factor model; and (M3) a bifactor model. In each model, items were specified to load only on their hypothesized latent factor, with cross-loadings fixed to zero, consistent with standard confirmatory (as opposed to exploratory) factor analysis. Model fit was evaluated using CFI, TLI, RMSEA, and SRMR; acceptable fit was defined as CFI/TLI ≥ 0.95, RMSEA ≤ 0.08, and SRMR ≤ 0.08 [21].

### Reliability analysis

Internal consistency was assessed using Cronbach’s alpha and McDonald’s omega for each subscale and the total scale. Corrected item-total correlations and alpha-if-item-deleted statistics were computed in SPSS.

### Ethical considerations

This study was conducted in accordance with the Declaration of Helsinki. Ethical approval was obtained from the Jazan Research Ethics Committee, Jazan Health Cluster, Saudi Arabia (No. 2447), and permission was obtained from administrative authorities of the Endocrinology and Diabetes Centre. All participants provided informed consent prior to participation.

## Results

Table 1 depicts sociodemographic characteristics of the study participants. About 60% were females and slightly more than half of the participants resided in urban areas. About a third (33.8%) had an income of more than ten thousand Saudi Riyals per month (about 2,267 euros). About a quarter (27.4%) had experienced a single episode of hypoglycemia or ketoacidosis, and a similar proportion had 2–3 episodes of hyperglycemia in the last year. The vast majority (81.3%) were diagnosed more than 3 years ago.

**Table 1:** Sociodemographic and Clinical Characteristics of Study Participants (*N* = 299)

Variable	Category	*n* (%)
Gender	Male	122 (40.8%)
	Female	177 (59.2%)
Residence	Urban	158 (52.8%)
	Rural	141 (47.2%)
Education	Illiterate	18 (6.0%)
	High school/Pre-university	145 (48.5%)
	University and Postgraduate	136 (45.5%)
Occupation	Employed	181 (60.5%)
	Unemployed	118 (39.5%)
Marital Status	Married	106 (35.5%)
	Single	168 (56.2%)
	Divorced	20 (6.7%)
	Widow	5 (1.7%)
Family Income	< 5000 SAR	48 (16.1%)
	5000–10,000 SAR	150 (50.2%)
	> 10,000 SAR	101 (33.8%)
Hypoglycemia	Not at all	106 (35.5%)
	Once	79 (26.4%)
	2–3 times	80 (26.8%)
	> 3 times	34 (11.4%)
Hyperglycemia	Not at all	115 (38.5%)
	Once	52 (17.4%)
	2–3 times	82 (27.4%)
	> 3 times	50 (16.7%)
ketoacidosis	Not at all	209 (69.9%)
	Once	75 (25.1%)
	2–3 times	10 (3.3%)
	> 3 times	5 (1.7%)
Diagnosis Date	< 1 year	15 (5.0%)
	1–3 years	41 (13.7%)
	> 3 years	243 (81.3%)
**Variable**		**Mean ± SD**
Age (Years)		30.4 ± 13.4
BMI		24.2 ± 5.8
HbA1c (%)		9.5 ± 2.1
FBG (mg/dl)		198.67 ± 86.80

SAR: Saudi Riyals; BMI: Body Mass Index; HbA1c: Glycated Hemoglobin; FBG: Fasting Blood Glucose

Table 2 summarizes participants’ responses to the DSAS-1 items. Overall, the combined proportion of strongly disagree and disagree responses was approximately 50% across statements. Two items showed particularly high levels of disagreement (> 70%): perceptions that injecting insulin in public would be viewed as drug use, and feeling self-conscious about diabetes management tools. In contrast, the most endorsed statements (agree + strongly agree, > 47%) were related to perceived blame for having Type 1 diabetes and concerns about others’ reactions when performing diabetes management in public. Subscale total scores (M ± SD): Blame & Judgment = 17.06 ± 8.78 (range 6–30); Identity Concerns = 17.19 ± 8.93 (range 7–35); Treated Differently = 15.84 ± 8.09 (range 6–30); Total scale = 50.08 ± 25.30 (range 19–95).

**Table 2:** Participants’ Responses to statements of DSAS-1 (*N* = 299)

Sub-scale	Item	Response *n* (%)				
		Strongly Disagree	Disagree	Not Sure	Agree	Strongly Agree
**Blame & Judgment**						
BJ1	Some people make unfair assumptions about what I can and cannot do because of my Type 1 diabetes.	93 (31.1%)	44 (14.7%)	32 (10.7%)	75 (25.1%)	55 (18.4%)
BJ2	Some people think I need insulin because I have not looked after myself.	94 (31.4%)	51 (17.1%)	19 (6.4%)	83 (27.8%)	52 (17.4%)
BJ3	Because I have Type 1 diabetes, some people judge me if I eat sugary food or drinks (e.g. cakes, lollies, soft drinks).	97 (32.4%)	42 (14.0%)	18 (6.0%)	77 (25.8%)	65 (21.7%)
BJ4	Some people think I am irresponsible when my diabetes management is not “perfect.”	103 (34.4%)	37 (12.4%)	28 (9.4%)	78 (26.1%)	53 (17.7%)
BJ5	Some people assume that it is my fault I have Type 1 diabetes (e.g. I ate too much sugar, I could have prevented it).	98 (32.8%)	38 (12.7%)	16 (5.4%)	85 (28.4%)	62 (20.7%)
BJ6	Some people think that I brought Type 1 diabetes on myself.	100 (33.4%)	45 (15.1%)	25 (8.4%)	85 (28.4%)	44 (14.7%)
**Identity Concerns**						
IC1	I feel embarrassed about what people might think if I need help with a hypo.	94 (31.4%)	43 (14.4%)	16 (5.4%)	103 (34.4%)	43 (14.4%)
IC2	I feel worried about telling people I have Type 1 diabetes in case they react negatively.	99 (33.1%)	69 (23.1%)	11 (3.7%)	73 (24.4%)	47 (15.7%)
IC3	I feel self-conscious about all the tools I need to manage my Type 1 diabetes (e.g. insulin pen, pump, blood glucose meter).	133 (44.5%)	85 (28.4%)	11 (3.7%)	41 (13.7%)	29 (9.7%)
IC4	To avoid negative reactions, I do not tell people I have Type 1 diabetes.	130 (43.5%)	64 (21.4%)	15 (5.0%)	57 (19.1%)	33 (11.0%)
IC5	I feel embarrassed when I have to manage my Type 1 diabetes in public (e.g. check blood glucose, inject or bolus insulin, refuse food, eat extra food).	128 (42.8%)	72 (24.1%)	14 (4.7%)	49 (16.4%)	36 (12.0%)
IC6	If I were to inject insulin in public, people would think I was taking drugs.	162 (54.2%)	51 (17.1%)	22 (7.4%)	39 (13.0%)	25 (8.4%)
IC7	I worry what people will think if they see me injecting or bolusing insulin, or checking my blood glucose in public.	98 (32.8%)	46 (15.4%)	10 (3.3%)	100 (33.4%)	45 (15.1%)
**Treated Differently**						
TD1	Because I have Type 1 diabetes, I have been excluded by others from certain social events.	107 (35.8%)	55 (18.4%)	67 (22.4%)	29 (9.7%)	41 (13.7%)
TD2	Some people expect less of me because I have Type 1 diabetes.	95 (31.8%)	42 (14.0%)	24 (8.0%)	79 (26.4%)	59 (19.7%)
TD3	I have been discriminated against in the workplace because I have Type 1 diabetes.	111 (37.1%)	36 (12.0%)	76 (25.4%)	41 (13.7%)	35 (11.7%)
TD4	Some people think I am unreliable because I have Type 1 diabetes.	108 (36.1%)	35 (11.7%)	74 (24.7%)	54 (18.1%)	28 (9.4%)
TD5	I have been rejected by others (e.g. friends, colleagues, romantic partners) because of my Type 1 diabetes.	96 (32.1%)	48 (16.1%)	56 (18.7%)	57 (19.1%)	42 (14.0%)
TD6	Some people see me as a lesser person because I have Type 1 diabetes.	103 (34.4%)	40 (13.4%)	25 (8.4%)	82 (27.4%)	49 (16.4%)

Note. DSAS-1: Diabetes Stigma Assessment Scale-1; BJ: Blame & Judgments; IC: Identity Concerns; TD: Treated Differently

### Translation and adaptation outcomes

The stepwise translation process resulted in a conceptually accurate and culturally appropriate Arabic version of the DSAS-1.

### Forward translation

After receiving the forward translation, a discussion ensued. During the discussion facilitated by the principal investigator, some adjustments were made including:

- Some phrases that were difficult for participants to understand (e.g., “lesser person”) were replaced with more comprehensible equivalents while maintaining the intended meaning.

### Backward translation

The quality of the forward translation was confirmed by the semantic consistency of the backward translation with the original tool, supporting the quality of the forward translation.

### Clinical Review

Three of the authors with extensive experience in managing diabetes patients and two external family physicians confirmed the tool’s appropriateness for clinical use.

### Cognitive interview findings

Participants were able to comprehend and answer the items most of the time. Minor clarification was needed for:

- **Item 3** (“lesser person”): One or two participants requested explanation, but understood it after clarification.
- **Item 12** (“romantic relationships”): The phrase seemed to be culturally sensitive in a conservative community. It was replaced by the phrase “life partners”, which was assured to retain the original intended meaning.

### Final proofreading and corrections

The feedback provided by the language expert at the University of Khartoum contained some minor typographic and grammatical corrections. Corrections were adopted and did not affect the content of the translation. The Arabic translation is available on the site of Mapi Research Trust, the copyright holders (https://eprovide.mapi-trust.org/*).*

#### Reliability

Internal consistency was good across all subscales. The Blame and Judgment (BJ) subscale demonstrated the highest reliability (alpha = 0.972, omega = 0.972), followed by Treated Differently (alpha = 0.965, omega = 0.966) and Identity Concerns (alpha = 0.946, omega = 0.946). For the total 19-item scale, alpha = 0.985 and omega = 0.986. All corrected item-total correlations exceeded 0.70 (range: 0.715–0.924), and no item deletion would meaningfully improve subscale reliability. Full item-level reliability statistics are presented in Supplementary A (Table 1).

### Factor structure

Table 3 presents standardised factor loadings, standard errors, z-values, and R² for the three-factor model (M2). All loadings were statistically significant (*p* <.001) and ranged from 0.642 (IC6) to 0.947 (TD6). The BJ subscale showed the most uniform and consistently high loadings (0.905–0.939), followed by TD (0.846–0.947). The lowest loadings were observed within IC (0.642–0.943), with IC6 showing the weakest relationship with its factor (R² = 0.412).

**Table 3:** Standardised CFA Factor Loadings, Standard Errors, z-values, and R² for the Three-Factor Model (MLR; *N* = 299)

Item	Factor	Std. Loading	SE	z	*p*	*R*²
BJ1	Blame & Judgment	0.905	-	-	< 0.001	0.819
BJ2		0.924	0.037	27.46	< 0.001	0.854
BJ3		0.925	0.039	27.54	< 0.001	0.856
BJ4		0.939	0.037	28.82	< 0.001	0.882
BJ5		0.921	0.039	27.16	< 0.001	0.848
BJ6		0.931	0.036	28.01	< 0.001	0.866
IC1	Identity Concerns	0.912	-	-	< 0.001	0.832
IC2		0.884	0.039	25.05	< 0.001	0.781
IC3		0.717	0.044	16.23	< 0.001	0.514
IC4		0.783	0.043	19.05	< 0.001	0.613
IC5		0.805	0.042	20.13	< 0.001	0.648
IC6		0.642	0.047	13.61	< 0.001	0.412
IC7		0.943	0.034	30.46	< 0.001	0.889
TD1	Treated Differently	0.846	-	-	< 0.001	0.715
TD2		0.921	0.053	22.74	< 0.001	0.848
TD3		0.878	0.050	20.71	< 0.001	0.771
TD4		0.896	0.048	21.51	< 0.001	0.802
TD5		0.939	0.048	23.69	< 0.001	0.882
TD6		0.947	0.051	24.10	< 0.001	0.897

All loadings significant at *p* <.001. First item per factor fixed to 1.0 for identification (SE and z not estimated). R² = squared multiple correlation. BJ = Blame & Judgment; IC = Identity Concerns; TD = Treated Differently; MLR = maximum likelihood with robust standard errors

Table 4 presents fit indices for all three competing models. None of the three competing models achieved acceptable fit by conventional criteria (Table 4).

**Table 4:** Goodness-of-Fit Indices for Competing CFA Models (*N* = 299)

Model	χ²	df	*p*	CFI	TLI	RMSEA	SRMR
Acceptable threshold	-	-	> 0.05	≥ 0.95	≥ 0.95	≤ 0.08	≤ 0.08
M1: One-factor	2000.85	152	< 0.001	0.812	0.789	0.202	-
M2: Three-factor correlated (MLR)	1960.47	149	< 0.001	0.816	0.789	0.202	0.065
M3: Bifactor (MLR)	1240.09	133	< 0.001	0.888	0.855	0.167	-

MLR = maximum likelihood with robust standard errors. Acceptable thresholds: CFI/TLI ≥ 0.95; RMSEA ≤ 0.08; SRMR ≤ 0.08 (Hu & Bentler, 1999)

#### Known-groups validity

Preliminary evidence of construct validity was examined through known-groups comparisons (Supplementary Table 2). Female participants reported significantly higher stigma scores than males across the BJ (*p* =.041), IC (*p* =.014), and Total scale (*p* =.023). Urban residents reported higher Identity Concerns and Total stigma scores than rural residents (*p* =.019 and *p* =.043, respectively). Participants aged ≥ 50 years reported higher Identity Concerns scores than younger participants (*p* =.018). Stigma scores were consistently higher among participants with uncontrolled versus controlled HbA1c (> 7.0% vs. ≤ 7.0%), though this difference did not reach statistical significance for any subscale. Participants who had experienced any episode of hypoglycemia, hyperglycemia, or ketoacidosis in the past year reported significantly higher stigma scores than those with no such episodes, across all subscales (all *p* <.001).

## Discussion

This study addresses the gap of scarcity of instruments that measure T1D stigma. It provides researchers in Arabic-speaking communities with a translation, cultural adaptation, and psychometric evaluation of the Type 1 Diabetes Stigma Assessment Scale (DSAS-1-Ar). Results suggest that the instrument reliably captures meaningful stigma-related constructs. However, model fit indices were suboptimal. As such, this study should be interpreted as a first step towards a fully validated T1D stigma measure. Future research should include addressing convergent validity, test-retest stability, and model performance in more diverse Arabic-speaking samples.

In this translation to Arabic, all items loaded significantly onto their respective factors, with the strongest loadings (most > 0.90) observed for Treated Differently and Blame and Judgment and internal consistency was good (total α = 0.985, ω = 0.986). Preliminary construct validity evidence is encouraging. Model fit indices (χ², CFI, TLI, RMSEA, and SRMR), however, indicated suboptimal fit across all tested structural models, and near-unity inter-factor correlations suggest that the three subscales functioned largely as a single construct. A recent exploratory analysis showed relevant associations between diabetes stigma and self-reported clinical variables in our research sample, thereby providing preliminary evidence for construct validity [15]. Construct validity of the DSAS-1 was further supported by the known-groups analyses we presented in the current study. The pattern of findings: higher stigma in females, urban residents, and those with more diabetes-related complications (hyperglycemia, hypoglycemia and ketoacidosis) is theoretically coherent and consistent with the broader T1D stigma literature [13].

The lowest item loadings were found in the Identity Concerns subscale, consistent with concerns that were expressed during the translation process. One such item, “*If I were to inject insulin in public*,* people would think I was taking drugs*”, may be influenced by cultural context. In Saudi Arabia, illicit drug use carries substantial stigma and severe legal consequences, which may cause participants to respond based on the stigma of drug use itself rather than stigma specific to diabetes. The second item, “*I am self-conscious about all the tools I need to deal with type 1 diabetes*”, uses the term *self-conscious*, which lacks a direct Arabic equivalent. Some respondents may have misinterpreted the item as referring to knowledge or skill related to diabetes tools rather than social awareness or discomfort. These nuances suggest that these items may benefit from refinement in future applications to improve conceptual clarity. These are not minor translation issues; they may indicate differential item functioning or cultural non-equivalence within the IC subscale. We recommend that IC items, particularly IC3 (“*self-conscious*”) and IC6 (“*injecting = drug use*”), undergo cognitive retesting and potential item refinement before the DSAS-1-Ar is deployed in large-scale research. Similar difficulties in model fit, particularly within the Identity Concerns domain, have been noted in other adaptations of DSAS-1, including the Danish version [22], suggesting that some items are inherently sensitive to cultural and linguistic variation.

The strong internal consistency of the DSAS-1-Ar is an important strength of this adaptation. Total scale alpha = 0.985 and omega = 0.986 are among the highest reported for any DSAS adaptation to date, exceeding those of the original English validation [14] and the Danish adaptation [22]. All corrected item-total correlations exceeded 0.70. This indicates that the Arabic items are able to capture their intended constructs and that the adaptation preserved the semantic integrity of the scale.

Hansen et al. (2018) [22] noted that the initially specified three-factor model did not meet several recommended fit criteria in their Danish sample, with only RMSEA falling within acceptable limits. A similar situation was reported by Alzubaidi et al. (2022) [23] while conducting psychometric analysis of an Arabic translation of the closely related 19-item Type 2 Diabetes Stigma Assessment Scale (DSAS-2), whereby they encountered poor fit across all indices except for χ² in their confirmatory factor analysis. This was not fully resolved after allowing correlated residuals, which resulted in χ² and CFI meeting criteria, while other indices continued to indicate poor fit. This pattern mirrors our experience, where the a priori three-factor structure showed only partial support, with some indices meeting standards while others suggested room for improvement. Taken together, these similarities suggest that such challenges are not unique to our Arabic translation, but may reflect broader characteristics of the DSAS-1 measurement model, as well as linguistic nuances and cultural interpretations of stigma-related constructs.

### Limitations

This study has several limitations related to translation and psychometric evaluation. The sample was drawn from a single clinical setting reflecting a specific healthcare and sociocultural environment, which may limit the generalisability of the findings to the wider population of Arabic-speaking adults with type 1 diabetes. The study used a cross-sectional design, so consistency of findings over time cannot be guaranteed. The pronounced floor effect may have reduced statistical power to detect factor structure, and measurement invariance across subgroups was not assessed. All data were self-reported, which may have influenced how participants interpreted or responded to certain items. Further testing of the scale in more diverse samples is needed to confirm the stability of the factor structure and refine items that appear culturally sensitive. Various analyses that were not conducted in this study would be of value, including inspection of modification indices, testing for correlated residuals among similarly worded items, Content Validity Index calculation (CVI), Weighted Least Squares Mean and Variance-Adjusted (WLSMV) estimation with polychoric correlations, or exploratory structural equation model (ESEM) analyses allowing cross-loadings. The present study should be viewed as an early-stage exploration providing valuable insight into diabetes-related stigma in an under-researched population.

## Conclusions

There is a scarcity of validated instruments to measure T1D-related stigma in Arabic-speaking populations, and the present study addresses this gap by providing a rigorously translated and adapted Arabic version of the DSAS-1. Among 299 adults with T1D in Jazan, Saudi Arabia, all 19 items loaded significantly onto their hypothesized subscales, and internal consistency was excellent (total α = 0.985, ω = 0.986). Preliminary construct validity evidence is encouraging. However, global CFA fit indices, particularly for RMSEA, were suboptimal across all tested structural models, and very high correlations between factors (all close to unity) suggest that the three subscales mostly functioned as a single construct, likely reflecting the low stigma variability and pronounced floor effect observed. This study represents a first step, not a final validation. Future research can address convergent validity, test-retest stability, and model performance in more diverse Arabic-speaking samples.

## Supplementary Information

Below is the link to the electronic supplementary material.

Supplementary Material 1

Supplementary Material 2

## Data Availability

The datasets generated during this study are not currently publicly available as it is part of an ongoing project, but are available from the corresponding author on reasonable request.

## Abbreviations

T1D: Type 1 Diabetes
DSAS-1-Ar: Arabic version Type 1 Diabetes Stigma Assessment Scale
MENA: Middle East and North Africa
KSA: Kingdom of Saudi Arabia
DRS: Diabetes-Related Stigma
T2D: Type 2 Diabetes
HbA1c: Glycated Hemoglobin
(DSAS-1): Diabetes Stigma Assessment Scale-1
EDC: Endocrinology and Diabetes Centre
TD: Treated Differently
BJ: Blame and Judgment
IC: Identity Concerns
CFA: Confirmatory Factor Analysis
SPSS: Statistical Package for the Social Sciences
FBG: Fasting Blood Glucose
MLR: Maximum Likelihood estimation with Robust standard errors
CFI: Comparative Fit Index
TLI: Tucker–Lewis Index
RMSEA: Root Mean Square Error of Approximation
SRMR: Standardized Root Mean Square Residual
χ²: Chi-square statistic
SAR: Saudi Riyals
