# Supplementary material for: Translation, cultural adaptation, and psychometric evaluation of the arabic version of the type 1 diabetes stigma assessment scale (DSAS-1-Ar) among adults in Jazan, Saudi Arabia
Source: J Diabetes Metab Disord. 2026 Jul 24;25(2):208. doi: 10.1007/s40200-026-02022-2 (PMC13400507; doi:10.1007/s40200-026-02022-2)
Supplement: Supplementary file 1 — Supplementary Material 1 [file 40200_2026_2022_MOESM1_ESM.pdf]

Supplementary Table 2: Known Groups Comparisons: Total and Subscale Scores

Known Group	Outcome	Group 1 M (SD)	Group 2 M (SD)	W	p	d
**HbA1C (≤7.0% vs >7.0%)**	TDRS	Controlled (n=38) 45.74 (27.08)	Uncontrolled (n=261) 50.72 (25.02)	4365	0.227	-0.197
	Total_BJ	Controlled (n=38) 14.97 (8.76)	Uncontrolled (n=261) 17.36 (8.75)	4251.5	0.149	-0.273
	Total_IC	Controlled (n=38) 16.29 (9.91)	Uncontrolled (n=261) 17.32 (8.79)	4600	0.465	-0.115
	Total_TD	Controlled (n=38) 14.47 (8.65)	Uncontrolled (n=261) 16.04 (8.01)	4300.5	0.18	-0.193
**Hypoglycemia (None vs Any)**	TDRS	None (n=106) 42.38 (26.55)	Any (n=193) 54.32 (23.60)	7460	<0.001	-0.484
	Total_BJ	None (n=106) 14.32 (9.35)	Any (n=193) 18.56 (8.08)	7625.5	<0.001	-0.496
	Total_IC	None (n=106) 14.80 (9.22)	Any (n=193) 18.50 (8.51)	7500	<0.001	-0.422
	Total_TD	None (n=106) 13.25 (8.35)	Any (n=193) 17.26 (7.60)	7165.5	<0.001	-0.509
**Hyperglycemia (None vs Any)**	TDRS	None (n=115) 42.71 (27.16)	Any (n=184) 54.69 (22.96)	7737	<0.001	-0.486
	Total_BJ	None (n=115) 14.23 (9.40)	Any (n=184) 18.83 (7.89)	7717	<0.001	-0.541
	Total_IC	None (n=115) 15.04 (9.51)	Any (n=184) 18.53 (8.29)	7865	<0.001	-0.397
	Total_TD	None (n=115) 13.44 (8.58)	Any (n=184) 17.34 (7.41)	7515	<0.001	-0.494
**Ketosis (None vs Any)**	TDRS	None (n=209) 44.25 (24.51)	Any (n=90) 63.62 (21.78)	5278	<0.001	-0.816
	Total_BJ	None (n=209) 15.08 (8.71)	Any (n=90) 21.66 (7.06)	5531	<0.001	-0.797
	Total_IC	None (n=209) 15.21 (8.44)	Any (n=90) 21.79 (8.35)	5416	<0.001	-0.782
	Total_TD	None (n=209) 13.97 (7.82)	Any (n=90) 20.18 (7.01)	5244.5	<0.001	-0.818

Known Group	Outcome	Group 1 M (SD)	Group 2 M (SD)	W	p	d
**Residence (Urban vs Rural)**	TDRS	Urban (n=158) 53.03 (25.27)	Rural (n=141) 46.79 (25.02)	12626.5	0.043	0.248
	Total_BJ	Urban (n=158) 17.96 (8.64)	Rural (n=141) 16.05 (8.85)	12288	0.118	0.218
	Total_IC	Urban (n=158) 18.39 (9.08)	Rural (n=141) 15.84 (8.59)	12864	0.019	0.287
	Total_TD	Urban (n=158) 16.68 (8.09)	Rural (n=141) 14.89 (8.02)	12542	0.056	0.222
**Sex (Male vs Female)**	TDRS	Male (n=122) 46.22 (24.50)	Female (n=177) 52.75 (25.57)	9149.5	0.023	-0.26
	Total_BJ	Male (n=122) 15.83 (8.63)	Female (n=177) 17.90 (8.80)	9318.5	0.041	-0.238
	Total_IC	Male (n=122) 15.61 (8.50)	Female (n=177) 18.28 (9.08)	9018.5	0.014	-0.302
	Total_TD	Male (n=122) 14.79 (7.83)	Female (n=177) 16.56 (8.21)	9391	0.052	-0.221
**Age (<50 vs ≥50)**	TDRS	<50 (n=273) 49.38 (25.69)	≥50 (n=26) 57.42 (19.75)	2820	0.08	-0.32
	Total_BJ	<50 (n=273) 16.93 (8.98)	≥50 (n=26) 18.35 (6.18)	3300	0.55	-0.16
	Total_IC	<50 (n=273) 16.83 (8.98)	≥50 (n=26) 20.92 (7.57)	2562	0.018	-0.46
	Total_TD	<50 (n=273) 15.62 (8.20)	≥50 (n=26) 18.15 (6.55)	2826	0.082	-0.31

Note. M: Mean; SD: Standard Deviation; HbA1c: Glycated Hemoglobin; TDRS: Total Diabetes Related Stigma; BJ: Blame & Judgments; IC: Identity Concerns; TD: Treated Differently
