# Supplementary material for: Translation, cultural adaptation, and psychometric evaluation of the arabic version of the type 1 diabetes stigma assessment scale (DSAS-1-Ar) among adults in Jazan, Saudi Arabia
Source: J Diabetes Metab Disord. 2026 Jul 24;25(2):208. doi: 10.1007/s40200-026-02022-2 (PMC13400507; doi:10.1007/s40200-026-02022-2)
Supplement: Supplementary file 2 — Supplementary Material 2 [file 40200_2026_2022_MOESM2_ESM.pdf]

Supplementary Table 1: Item-level reliability statistics for the DSAS-1-Ar subscales

Item	Subscale	M	SD	Corrected Item-Total r	Alpha if Deleted
BJ1	Blame & Judgment	2.85	1.54	0.884	0.969
BJ2		2.83	1.54	0.903	0.967
BJ3		2.90	1.60	0.922	0.966
BJ4		2.80	1.56	0.924	0.965
BJ5		2.92	1.60	0.915	0.966
BJ6		2.76	1.52	0.906	0.967
IC1	Identity Concerns	2.86	1.52	0.800	0.940
IC2		2.67	1.52	0.863	0.934
IC3		2.16	1.37	0.821	0.938
IC4		2.33	1.46	0.870	0.934
IC5		2.31	1.46	0.873	0.933
IC6		2.04	1.38	0.715	0.946
IC7		2.83	1.54	0.800	0.940
TD1	Treated Differently	2.47	1.41	0.842	0.963
TD2		2.88	1.57	0.887	0.959
TD3		2.51	1.41	0.880	0.959
TD4		2.53	1.38	0.911	0.956
TD5		2.67	1.45	0.907	0.956
TD6		2.78	1.55	0.906	0.956

Note. M = Mean; SD: Standard Deviation; DSAS-1-Ar: Arabic version Type 1 Diabetes Stigma Assessment Scale; r: Corrected item-total correlation. Subscale alphas: BJ $\alpha = .972$ ($\omega = .972$); IC $\alpha = .946$ ($\omega = .946$); TD $\alpha = .965$ ($\omega = .966$); Total $\alpha = .985$ ($\omega = .986$).
